# Accuracy of Motor Error Predictions for Different Sensory Signals

**DOI:** 10.3389/fpsyg.2018.01376

**Published:** 2018-08-07

**Authors:** Michael Joch, Mathias Hegele, Heiko Maurer, Hermann Müller, Lisa K. Maurer

**Affiliations:** Neuromotor Behavior Laboratory, Department of Psychology and Sport Science, Justus Liebig University Giessen, Giessen, Germany

**Keywords:** EEG, error negativity, feedback-related negativity, error prediction, reinforcement learning, forward model

## Abstract

Detecting and evaluating errors in action execution is essential for learning. Through complex interactions of the inverse and the forward model, the human motor system can predict and subsequently adjust ongoing or subsequent actions. Inputs to such a prediction are efferent and afferent signals from various sources. The aim of the current study was to examine the impact of visual as well as a combination of efferent and proprioceptive input signals to error prediction in a complex motor task. Predicting motor errors has been shown to be correlated with a neural signal known as the error-related negativity (Ne/ERN). Here, we tested how the Ne/ERN amplitude was modulated by the availability of different sensory signals in a semi-virtual throwing task where the action outcome (hit or miss of the target) was temporally delayed relative to movement execution allowing participants to form predictions about the outcome prior to the availability of knowledge of results. 19 participants practiced the task and electroencephalogram was recorded in two test conditions. In the *Visual* condition, participants received only visual input by passively observing the throwing movement. In the *EffProp* condition, participants actively executed the task while visual information about the real and the virtual effector was occluded. Hence, only efferent and proprioceptive signals were available. Results show a significant modulation of the Ne/ERN in the *Visual* condition while no effect could be observed in the *EffProp* condition. In addition, amplitudes of the feedback-related negativity in response to the actual outcome feedback were found to be inversely related to the Ne/ERN amplitudes. Our findings indicate that error prediction is modulated by the availability of input signals to the forward model. The observed amplitudes were found to be attenuated in comparison to previous studies, in which all efferent and sensory inputs were present. Furthermore, we assume that visual signals are weighted higher than proprioceptive signals, at least in goal-oriented tasks with visual targets.

## Introduction

Previous research suggests that prediction represents a general framework underlying many perceptual and motor processes (Yuille and Kersten, 2006; Friston and Kiebel, 2009; Bubic et al., 2010; Bar, 2011; Clark, 2013). Predictive motor control is closely connected to the concept of internal models. It is currently thought that the motor system uses two forms of internal models: inverse models and forward models (Jordan and Rumelhart, 1992; Miall and Wolpert, 1996; Wolpert and Flanagan, 2001; Shadmehr et al., 2010). Inverse models relate intended action goals to the motor commands to achieve those goals and thus generate motor commands sent to the effectors to produce the intended sensory consequences. Forward models represent the inverse direction of causality relating the motor commands to the resultant sensory consequences and thus predict the future state of the sensorimotor system. These predictions can serve a multitude of functions, such as compensating for delays and noise in neural signal transduction, distinction between self and others, attenuation of self-produced sensory reafferences, or facilitating executive functions such as response inhibition (Mirabella, 2014). In this study, we seek to explore predictive mechanisms underlying performance monitoring and error perception in the course of motor learning.

In the context of motor learning, humans constantly need to process information from the environment and from internal sources to improve and maintain performance. In order to improve the performance in a motor task and learn from previous errors, we have to be able to detect that the intended action goal has not been achieved and subsequently attribute this failure to a cause (Holroyd and Coles, 2002). Predictions generated by an internal forward model can support error attribution. If errors were predicted by the forward model during action execution, these errors would most likely be due to internal causes (e.g., inappropriately selected motor commands). On the other hand, if the intended action goal was not achieved and no error was predicted by the forward model throughout the movement, errors should be attributed to external perturbations (e.g., the wind has changed the trajectory of the ball during a free kick in soccer) and should not trigger subsequent adjustments to the motor commands as long as the perturbations are unsystematic. Another advantage of error prediction pertains to the learning of sensorimotor skills that are characterized by a temporal separation of action execution and the perception of action outcome. Based on the observation that delays in the availability of feedback about the outcome of an action attenuate sensorimotor learning (Kitazawa et al., 1995; Kitazawa and Yin, 2002; Brudner et al., 2016; Schween and Hegele, 2017), predicting errors before they actually occur could support learning, as the error signal would be closer in time to movement execution. Thus, forward model predictions can offer valuable information to adequately adapt movements during motor learning.

The importance of forward model predictions in learning and their development are highlighted by both computational models (Jordan and Rumelhart, 1992; Wolpert et al., 1998; Haruno et al., 2001) and empirical studies (Flanagan et al., 2003; Tseng et al., 2007; Shadmehr et al., 2010). Neurophysiological approaches, more specifically the analysis of event-related potentials (ERPs) in the electroencephalogram (EEG), provide more detailed insights into the time course of error processing and prediction. Two of the most prominent ERPs in the EEG with respect to error processing are the error-related negativity (Ne/ERN; Falkenstein et al., 1991b; Gehring et al., 1993) and the feedback-related negativity (FRN; Miltner et al., 1997). The sources of both potentials are primarily located in the medial prefrontal cortex including the anterior cingulate cortex (ACC; Location Ne/ERN: Dehaene et al., 1994; Mathalon et al., 2003; Holroyd et al., 2004; Debener et al., 2005; Location FRN: Luu et al., 2003). Furthermore, both potentials are present in situations in which erroneous movements or incorrect motor responses to a stimulus result in a failure of achieving the desired movement outcome. However, it is important to clearly distinguish between Ne/ERN and FRN as they reflect error processing at different time points throughout an action. The FRN can be observed after feedback about the action outcome is available, i.e., it reflects a REACTION to this outcome feedback. In contrast, the Ne/ERN is manifested shortly after movement onset and, importantly, prior to feedback about action outcome. In these cases, the Ne/ERN reflects a PREDICTION of an event in the future (i.e., an upcoming error). The onset times of the Ne/ERN can vary, depending on the type of motor task, from 80 to 100 ms after movement onset in choice-reaction time tasks (Falkenstein et al., 1991a; Gehring et al., 1993) to 200–350 ms in motor tasks composed of multiple submovements (Anguera et al., 2009; Maurer et al., 2015; Joch et al., 2017). However, as long as the Ne/ERN is related to the action outcome and emerges prior to external feedback about action outcome, it seems reasonable to consider the Ne/ERN as a correlate of predictive error processing, while the FRN in response to outcome feedback is a correlate of postdictive error processing. In this article, we will focus on the question whether the availability of different sensory signals modulates predictive error perception.

The functional significance of the Ne/ERN has been discussed in light of post-response conflict monitoring (Yeung et al., 2004), reinforcement learning (Holroyd and Coles, 2002), and surprise because of the non-occurrence of predicted events (Alexander and Brown, 2011). Regardless of whatever explanation might hold, all of these functions require prediction in the absence of external outcome feedback. Only with prediction about an outcome, it is possible to process information about a deviation to this expected outcome, to detect an error or to evaluate a conflict about the correct outcome. Since the Ne/ERN emerges prior to the availability of external outcome feedback, it is reasonable to assume that the Ne/ERN arises from the output o^ of a predictive model *P* (e.g., an internal forward model) on the basis of several input signals *I* and their respective weights w[P(I1⋅w1⋅⋅⋅In⋅wn)o^]. The Ne/ERN can be interpreted as a correlate of the comparison between predicted outcome and intended outcome indicating an upcoming error. Input signals to the predictive model might be provided by efferent information (e.g., via efference copy) and by afferent information about the environment, the movement execution, and the movement outcome (e.g., via visual or proprioceptive signals).

Feedback about the final outcome of an action is not the only source of error information available. In a throwing task we see and feel our hand moving and we see the thrown object flying toward the target clearly before we observe the final result [hit or miss]. Previously, we defined continuous (visual) information about the immediate movement effect (the flying ball), which is available prior to outcome feedback (hit or miss), as action effect monitoring (Maurer et al., 2015). In a recent study, Joch et al. (2017) showed that error prediction is possible in the absence of action effect monitoring: a Ne/ERN signal was observed in a target-oriented ball-throwing task even when information about the ball trajectory toward the target was not shown. Yet, the Ne/ERN amplitude was noticeably smaller compared to the control condition where effect monitoring was possible (Maurer et al., 2015). The attenuation of the Ne/ERN can be interpreted as an increase in uncertainty about o^ when restricting input information to *P*.

The aim of the present study was to estimate the contribution of other afferent and efferent signals to error prediction as quantified by the amplitude of the Ne/ERN. We used the same semi-virtual ball throwing task (Skittles) as described in Maurer et al. (2015) and Joch et al. (2017) and removed (a) visual information about movement execution as well as action effect monitoring in one condition and (b) proprioceptive and efferent signals related to movement execution in another condition. As a result, we expected a further decrease of the Ne/ERN amplitude as an expression of increasing prediction uncertainty due to a reduced number of input signals to the predictive model. Since the additional removal of input signals could diminish the effects to the extent that the signal becomes smaller than the noise in the EEG signal, we sought to validate our results by comparing effects on the Ne/ERN with effects on the FRN with the rationale being as follows: based on the notion of the Ne/ERN being the first neural indicator of a motor error (Holroyd and Coles, 2002; Stahl, 2010), cases when an error of the movement outcome occurs and can be predicted based on internal information, should render external outcome information [i.e., knowledge of results (KRs) feedback] less relevant for the motor system. In other words, if the error was already predicted by the internal prediction model (i.e., higher Ne/ERN amplitude), the motor system should not be “surprised” to perceive error feedback (resulting in a lower FRN amplitude). This complementary behavior of Ne/ERN and FRN has been shown in several studies (e.g., Holroyd and Coles, 2002; Pietschmann et al., 2008). Thus, we will take advantage of this reciprocal behavior of Ne/ERN and FRN to better understand the absence of a significant fronto-central negativity in either condition.

## Materials and Methods

### Participants

Participants of the current EEG study were recruited from the student population of the Justus Liebig University Giessen. The sample consisted of 19 participants (four males) with an average age of 21.7 years (*SD* = 4.2 years). Participants received course credit and had the chance to win up to 30 € by participating in the experiment, which was conducted in accordance with the ethical standards laid down in the Declaration of Helsinki. The protocol was approved by the Ethical Review Board of the Justus Liebig University Giessen.

### The Semi-Virtual Throwing Task

Participants practiced a semi-virtual version of a British pub game called *Skittles*. In the real game, a ball is attached to the top of a post by a string. The player throws the ball in order to hit one or multiple target skittles on the opposite side of the post. In the semi-virtual adaptation of Skittles, this setup is displayed on a computer monitor (size: 15-inch, format: 3:4; model: AOC 919Va2, screen resolution: 1024 × 768 Pixel) from a bird’s-eye view. On the computer screen, participants could see a green BALL (radius on display = 2.5 mm) which had to be thrown around a blue center POST (radius on display = 12.5 mm) to hit a red TARGET object (radius on display = 2.5 mm). The ball’s trajectory was determined by the simulated physics of the task (Müller and Sternad, 2004) and described an elliptic path around the post. In the model, the relevant objects were defined as follows: center POST (radius = 0.25 m; position: x = 0.0 m, y = 0.0 m), TARGET (radius = 0.05 m; position: x = 0.35 m, y = 1.0 m), BALL (radius = 0.05 m).

**FIGURE 1:**
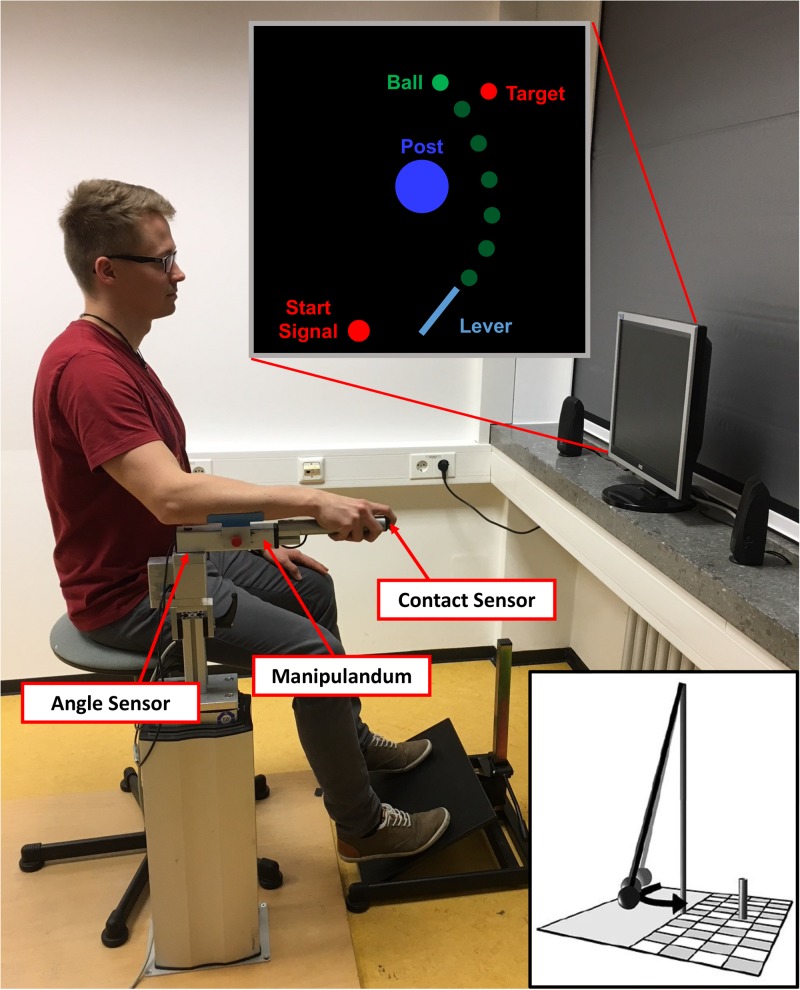
Setup of the virtual adaptation of the Skittles task. The participant uses the manipulandum to throw the green ball with a horizontal rotational movement. The ball travels with an elliptical trajectory around the central post and toward the target. The distance between the manipulandum and the computer screen was one meter. The depicted participant gave written informed consent for the publication of this picture. The little insert depicts a symbolic illustration of the real version of Skittles.

To throw the ball, participants used a metal lever (see **Figure 1**). They sat on a stool and rested their arm on a foam pad attached to the lever. The lever could be rotated within the horizontal plane around a vertical rotation axis located approximately under the participant’s elbow joint. The distance between the participants’ eyes and the computer screen was 1 m. A contact sensor was placed at the tip of the lever so that when participants placed their index finger on the sensor, the virtual ball was picked up and visually attached to a virtual equivalent of the lever (length: 0.4 m, position of the fixed end: x = 0.0 m, y = -1.5 m). Then, they rotated the lever clockwise and released the ball at any time during the movement by lifting their finger off the contact sensor. Because fast and rhythmic executions of subsequent trials could be a confounding factor of the results, we introduced a constant foreperiod before a Go-Signal appeared in the screen instructing the participants not to initiate their movement before its onset (see **Figure 1**, Start Signal). In detail, at the start of each trial, participants had to move the tip of the lever into a red circle positioned to the left of the fixed end of the lever (corresponding with a 0° lever position in the physical model). When the tip of the virtual lever reached the red circle, it immediately turned yellow and, subsequently, green when the lever was held at least 1 s within that circle. The green circle was used as a cue that the subjects were now free to move at any time. Note that participants did not start the throwing movement as a reaction to the green signal; it merely signaled that they were allowed to commence the movement at any time after the start circle had turned green.

The actual trajectory and thus the final outcome of the throwing movement was defined by angle of the lever and velocity of the BALL at the moment of its release. The feedback given during and after a trial depended on the experimental phase and condition. For more details about the experimental conditions see section “Study Design and Experimental Conditions”. Task performance was quantified by measuring the number of hits in relation to the total number of executed trials (i.e., hit rate).

The general task instruction was given in a standardized way at the beginning of the study, whereas condition specific instructions were provided directly before a specific condition started. To keep the participants motivated throughout the whole experiment, the individuals with the three highest target hit rates were rewarded with 30 € for the first, 20 € for the second and 10 € for the third place.

### Study Design and Experimental Conditions

**FIGURE 2:**
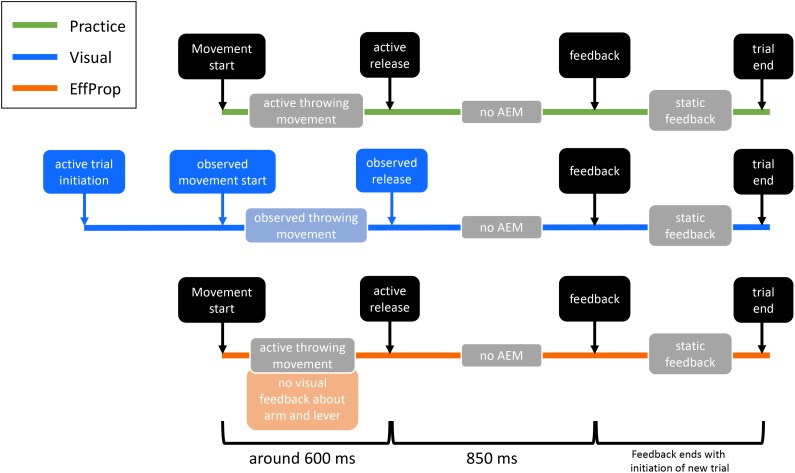
Illustration of the differences between practice and the two experimental conditions (*Visual, EffProp*). Shown are, one below the other, the temporal events of one trial in each condition. Similar action event are depicted in black. The lighter colored boxes represent information sources for error processing (AEM = action effect monitoring). Differences are marked with the corresponding color (green = practice, blue = *Visual* condition, orange = *EffProp* condition).

The study consisted of six sessions on six separate days. The first four sessions were used as practice/training sessions and the last two were experimental sessions, in which the motor task had to be executed under different conditions by the participants while EEG was measured. The duration of the practice sessions was approximately 30 min each and the EEG sessions had a duration of 1.5–2 h each. In order to improve the performance in the motor task (i.e., improve the hit rate), 400 trials had to be executed at every practice session (1600 practice trials in total). In addition, each EEG session consisted of 430 trials (see **Figure 2**).

#### Practice Phase (Sessions 1–4)

Since displaying the ball’s trajectory toward the target influences the neural prediction process (Joch et al., 2017), it was incrementally reduced during the practice sessions. For the first 200 trials of session 1, the ball trajectory was displayed starting immediately after the release of the ball from the virtual lever. In addition to this immediate dynamic feedback, a static depiction of the entire ball trajectory as well as a feedback about the action outcome were displayed on the screen 850 ms after ball release. In detail, the static feedback consisted of simultaneously depicted ball positions at temporally equidistant moments of ball flight along the trajectory of the actual trial (see **Figure 1**). Regarding outcome feedback, a collision sound was played for the hit trials, the target object was knocked out of its position, and the German word for “hit” (“Treffer”) was displayed in green on the computer screen. In case of an error, participants received the feedback “Unfortunately a miss” (“Leider vorbei”) written in red. Outcome feedback information was delayed by 850 ms because this was the average time it took the ball to reach the vicinity of the target object (calculated based on preliminary data). The static trajectory and the outcome feedback was presented throughout all four practice sessions. In contrast, the dynamic display of the ball flight was decreased by 33% every 100 trials over the second part of day 1. On day 2, participants started with 66% dynamic ball flight display, which was again decreased every 100 trials by 33%. As a result, they executed the last 200 trials of day 2 with 0% dynamic ball flight information. In the 0% condition, the ball was masked at the moment of ball release and participants exclusively received the static feedback and the result-feedback after 850 ms. Practice sessions 3 and 4 as well as the experimental sessions were conducted with 0% ball flight trajectory.

#### Visual (Session 5)

In session 5, participants passively observed trials on the computer screen. Hence, in this session, error prediction could solely be based on the visual input signals that were available until the observed ball release. For motivational reasons and to keep participants from forgetting the skill, the observation trials (*O*) were interspersed by normal execution trials (*E*), where the ball had to be actively thrown in order to hit the target analog to the practice sessions. In the observation blocks, subjects started an observation trial by directing the manipulandum and thus the virtual lever toward the starting position (red circular area) located at a 90° angle of the virtual lever. The observation trial started as soon as participants lifted the index finger off the contact sensor within the starting position. Participants saw the throwing movement of the virtual lever until the release of the ball but not the ball flying toward the target. After 850 ms, they received result-feedback in form of the static ball trajectory (analog to the practice session). The presented trials were taken from the participant’s practice phase (without their knowledge) and chosen so that the total number of observed trials were composed of 50% hits and 50% misses. Session 5 started with 20 execution trials to re-familiarize participants with the task. Afterward, observation trials and execution trials were alternatively conducted in blocks of 20 trials (*O*) and 10 trials (*E*), respectively. This block-wise condition switching was conducted until 280 observation trials had been recorded (i.e., the observation session ended with block *O*).

#### EffProp (Session 6)

On the sixth and last session of the study, participants completed the task without visual display of the movement. We removed any visual information about the effectors and the lever. In detail, manipulandum and throwing arm of the participant were covered by a horizontal board. The virtual lever on the computer screen was masked as soon as participants started their throwing movement. We determined the movement start by means of the angular velocity of the lever. Whenever the angle velocity exceeded 50°/s after being in the starting position, the movement was classified as started. Altogether, the participants had to execute 430 throws on the last day. There was a drop out of two participants in the *EffProp* condition due to technical changes.

### EEG Data Acquisition and Preprocessing

Acquisition of EEG data started on day 5. Furthermore, an electrooculogram (EOG) was conducted to measure eye movements (e.g., blinks). EOG electrodes were placed above and below the right eye and on the external canthi of both eyes. For the recordings, we used a 16 channel AC/DC amplifier with Ag/AgCl active scalp electrodes (V-Amp, Brain Products GmbH, Gilching, Germany). The position of the electrode was set according to the international 10–20 system (Klem et al., 1999). The actual positioning was done using the actiCAP electrode cap by Brain Products. Specifically, we used the electrodes F3, Fz, F4, FCz, C3, Cz, C4, P3, Pz, P4 and placed the ground electrode on the Fpz position. For signal reference, we used two electrodes, one online and one offline reference. The online reference electrode was placed on the left mastoid. The offline reference electrode was placed on the right mastoid. This electrode was used for offline re-referencing, hence, an average of both reference electrodes was used for further analyses. Electrodes impedances were held below 15 kΩ. The data was recorded using a 500 Hz acquisition frequency.

After data acquisition, EEG and EOG data were preprocessed offline using the Brain Vision Analyzer 2.1. software. First, the signals were filtered using a Butterworth filter with a low cut-off frequency of 0.2 Hz and a high cut-off of 30 Hz. To correct for ocular artifacts, we applied the ocular correction algorithm of the Analyzer 2.1 software, which is based on the Infomax Independent Component Analysis (ICA; Makeig et al., 1996, 1997). To calculate the ICA components, only EEG activity around blinks was fed into the ICA algorithm. Blinks were detected using the mean slope algorithm by Gratton et al. (1983). After visual inspection of the components, the component(s) explaining more than 30% of the eye movements were then removed from all other EEG activity.

After EOG correction, the signal was segmented. The size of the segments was different for the two experimental conditions. In the *Visual* condition, each segment began 600 ms before the observation trial was initiated by the participant. The end of the segment was set 2800 ms after the start of the segment. Hence, each segment included the following events: trial start, virtual ball release, and outcome feedback presentation. In condition *EffProp*, the segment started 600 ms before the subject released the ball in the throwing motion and it ended 2200 ms after the segment’s start. Each segment was manually controlled for remaining artifacts.

### Skittles Data Preparation

The electrophysiological potentials of interest, the Ne/ERN and FRN, typically emerge when an incorrect response to a stimulus is executed. Therefore, target hits and target misses had to be separated for further analyses. To do so, the minimal distance between the center of the thrown ball and the center of the target was measured yielding a distance (*d*) value. In the underlying physical model, ball and target both had a radius of 5 cm. Hence, trials with a *d* value greater than 10 cm were classified as misses. Trials in which the center post was hit were excluded from the analysis. Because close hits/misses could blur the neural signatures of hits and errors, we classified only trials with *d* ≤ 7 cm as hits and trials with *d* ≥ 12 cm as errors. In addition, hit rates for every session were calculated to measure the performance in the Skittles task. Task performance is assumed to be related to the quality of the internal forward model of the task (Jordan and Rumelhart, 1992).

### Statistical Analysis

For the statistical analyses, we used Mathworks MATLAB R2016a. To be able to statistically analyze the electrophysiological data, we conducted a mean amplitude analysis of the FCz segments that resulted from the data pre-processing. For this, a baseline corrected difference curve for every participant’s average hit and error curve was calculated. For baseline correction, we used the time interval between ball release and the effect window for the Ne/ERN (see below) as the baseline interval (i.e., 0–200 ms). The data of the difference curves were then averaged over *a priori* set effect windows (EffW) for the Ne/ERN (200–350 ms after ball release; EffW_ERN_) and the FRN (150–350 ms after feedback; EffW_FRN_) to yield a mean amplitude for EffW_ERN_ and EffW_FRN_ for each participant. Averages of the mean amplitudes can be found in **Table 1** (Δ*MeanAmpl.*). Note that the EffW_ERN_ in the *Visual* condition was set to 200–350 ms after the *observed* ball release (as opposed to the active ball release in the practice sessions and in *EffProp*). The resulting mean amplitudes were tested with a one-sample *t*-test using a test value of zero. To confirm the results from classical inference statistics, we used a Bayesian inference approach to calculate Bayes factors (*BF*) that can be interpreted as the amount of evidence for the null-hypothesis before versus after seeing the data (Verdinelli and Wasserman, 1995). The computation of the Bayes factors was done in JASP 0.8.2.0 and separately for both effect windows and both conditions. The size of the *BF*s are interpreted according to Raftery (1995).

**Table 1:** Overview of electrophysiological results.

	*Visual*		*EffProp*	
		
	EffW_ERN_	EffW_FRN_	EffW_ERN_	EffW_FRN_
Δ*MeanAmpl.*	-0.6 μV	-3.4 μV	0.05 μV	-5.6 μV
Δ*PeakAmpl.*	-1.8 μV	-7.9 μV	-1.6 μV	-10.7 μV
*p*	0.01	<0.001	0.55	<0.001
*Effect size d*	0.57	1.49	0.03	1.15
*BF*_10_	5.3	1063	0.21	170


To describe the ERPs in more detail, peak amplitudes were calculated for every participant within both effect windows. The peak amplitude was defined as the minimum activation (since Ne/ERN and FRN are negative potentials) in the corresponding effect window. The peak amplitudes of the participants were then averaged to yield average peak amplitudes (Δ*PeakAmpl.* in **Table 1**). Note that Δ*PeakAmpl.* can differ from the peak of the difference curves shown in the electrophysiological result figures because there the difference curves represent the difference between grand average curves of hits and misses respectively (averaged over all participants’ mean curves).

## Results

### Behavioral Results

**FIGURE 3:**
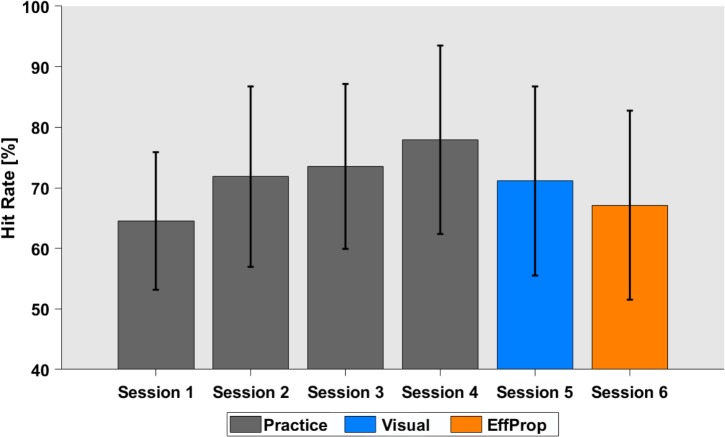
Task performance in hit rates over the four practice sessions (gray), the *Visual* (blue), and the *EffProp* condition (orange). Error bars represent standard deviations.

Participants practiced the Skittles task for four sessions on four separate days (400 trials per session) before EEG recordings were conducted in sessions 5 and 6. During the practice sessions, participants were able to improve task performance (quantified by the hit rate) from 64.6% (*SD* = 11.4%) in session 1 to 77.9% (*SD* = 15.6%) in session 4 [see **Figure 3**; *F*(3,88) = 3.657; *p* = 0.01].

The hit rate for the execution trials that alternated with the observation trials in the *Visual* condition was 71.1% (*SD* = 15.3%). The average hit rate slightly dropped in the last (*EffProp*) session to a hit rate of 67.1% (*SD* = 18.8%). However, this difference was not significant [*t*(16) = 1.18; *p* = 0.25].

### Electrophysiological Results

#### Condition: Visual

In the *Visual* condition, participants did not actively throw the ball toward the target but passively observed trials on the computer monitor. This way, the prediction model had access to visual signals of the lever movement while efferent as well as proprioceptive signals were unavailable.

**FIGURE 4:**
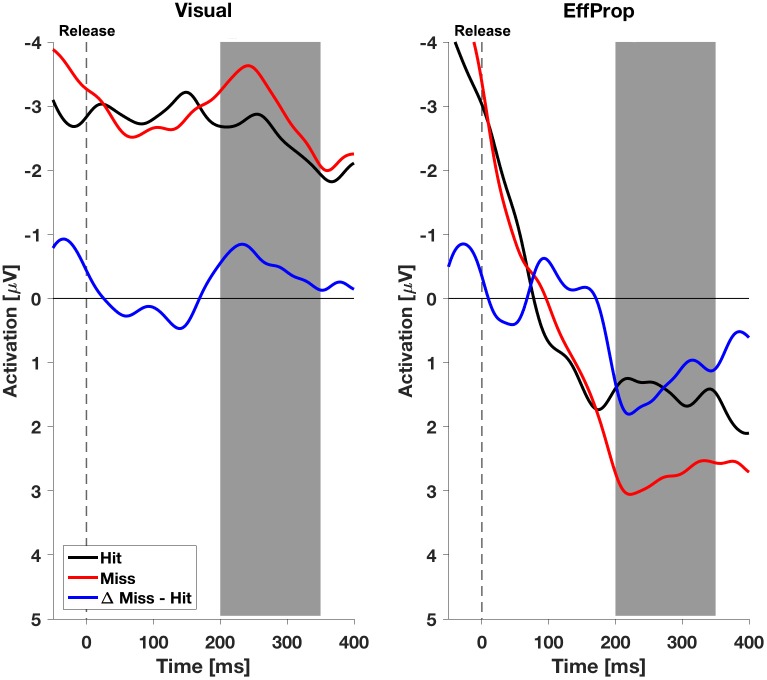
Electroencephalogram signals in the effect window EffW_ERN_, 200–350 ms after ball release for both experimental conditions (*Visual* and *EffProp*). Ball release is indicated with the broken line. As feedback was shown 850 ms after ball release, the gray shaded interval represents the time window for error prediction.

**FIGURE 5:**
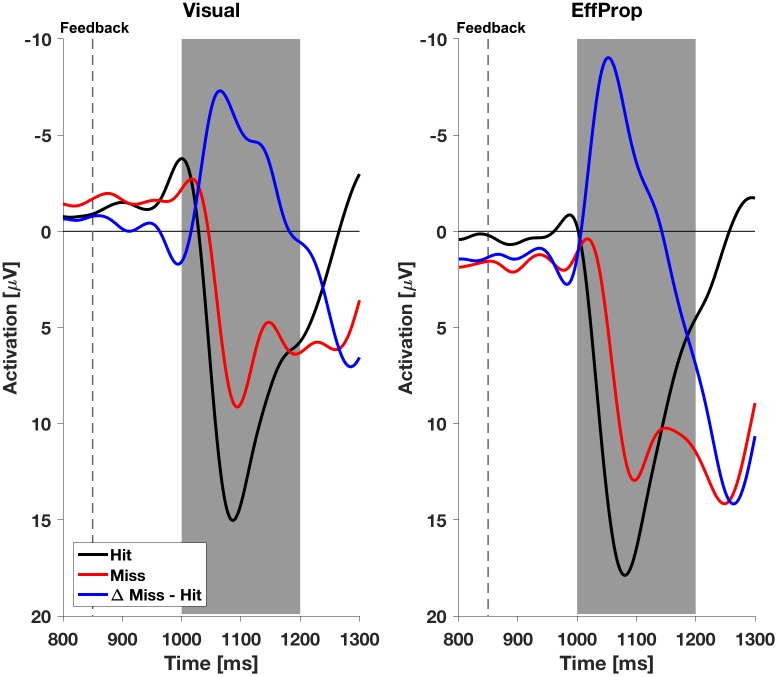
Electroencephalogram signals with respect to feedback in both experimental conditions (*Visual* and *EffProp*). The broken line represents the time terminal result feedback was provided. The gray shaded interval represents the time window for error postdiction.

We found a significant effect within the Ne/ERN effect window [*t*(17) = -2.4; *p* = 0.01; *d* = 0.57] determined by the mean amplitudes (Δ*MeanAmpl.*_ERN_ = -0.6 μV; *CI*_95%_ = [-1.18 μV, -0.08 μV]) of the difference curves (misses minus hit trials; see **Figure 4**, left). This result is supported by a Bayesian inference approach yielding a Bayes Factor of *BF*_10_ = 5.3 (corresponding to a positive evidence after Raftery, 1995). Furthermore, the ERP is characterized by a difference signal peak amplitude (Δ*PeakAmpl.*_ERN_) of -1.8 μV. In reaction to negative result-feedback, we observed a highly significant negative deflection (Δ*MeanAmpl.*_FRN_ = -3.4 μV; *CI*_95%_ = [-4.55 μV, -2.28 μV]) within the FRN effect window [*t*(17) = -6.3; *p* < 0.001; *d* = 1.49; *BF*_10_ = 1063] (i.e., very strong evidence; **Figure 5**, left). The measured peak amplitude was Δ*PeakAmpl.*_FRN_ = -7.9 μV.

#### Condition: EffProp

In the condition *EffProp*, participants had to actively throw the ball, but no visual information about movement execution and ball flight was available (i.e., the internal prediction model had access to proprioceptive and the efference copy signals, but not to visual signals). We found an average mean amplitude of Δ*MeanAmpl.*_ERN_ = 0.05 μV (*CI*_95%_ = [-0.80 μV, 0.90 μV]). There was no significant effect in the mean amplitudes of the difference curves within the preset Ne/ERN effect window [*t*(16) = 0.13; *p* = 0.55; *d* = 0.03; **Figure 4**, right]. In line with this result, the Bayesian analysis revealed more evidence for the null hypotheses in the data than for the alternative hypothesis (*BF*_10_ = 0.21). Furthermore, the measured peak amplitude of the difference curve within EffW_ERN_ was Δ*PeakAmpl.*_ERN_ = -1.6 μV.

Participants reacted with a sharp negative deflection to negative result-feedback (Δ*MeanAmpl.*_FRN_ = -5.6 μV; *CI*_95%_ = [-8.12 μV, -3.10 μV]) within the preset effect window EffW_FRN_ [*t*(16) = -4.73; *p* < 0.001; *d* = 1.15; *BF*_10_ = 170] (i.e., very strong evidence; **Figure 5**, right). The observed potential had a peak amplitude of Δ*PeakAmpl.*_FRN_ = -10.7 μV. An overview of the measured values in both conditions can be found in **Table 1**. Figures of the grand averages of all recorded electrodes can be found in **Supplementary Figures S1**–**S4**.

#### Visual vs. EffProp

**FIGURE 6:**
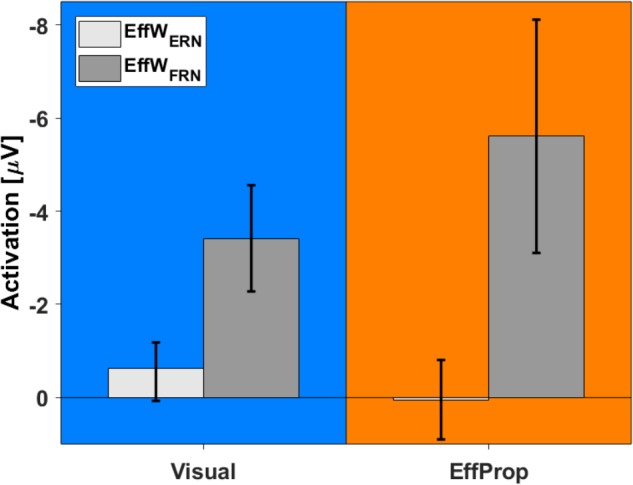
Comparison between activations found in *Visual* (blue) and *EffProp* (orange) with regard to the mean amplitude of the differences curves (signals measured in error trials minus signals measured in hit trials). Error bars represent 95% confidence intervals.

In addition to the intra condition testing, we tested if there were differences between the mean values for the *Visual* and *EffProp* conditions (see also **Figure 6**). Comparing the mean amplitude values of *Visual* and *EffProp* in the effect window for the error prediction (EffW_ERN_), we found a difference of 0.65 μV. However, the significance level was slightly missed [*t*(33) = -1.44; *p* = 0.08; *d* = 0.25]. In the Bayesian analysis, updating the prior distribution with the data revealed slightly more evidence for the alternative hypothesis (difference in mean amplitudes between *Visual* and *EffProp*) than for the null hypothesis [*BF*_10_ = 1.99 (i.e., weak evidence); *median effect* = 0.38].

With respect to the result-feedback reaction (EffW_FRN_), we found significantly larger mean ERP amplitudes (i.e., stronger negative deflections) in the *EffProp* condition compared to the *Visual* condition [*t*(33) = 1.77; *p* = 0.047; *d* = 0.31; see also **Figure 5**]. This observation is supported by the conducted Bayesian analysis [*BF*_10_ = 2.77 (i.e., weak evidence); *median effect* = 0.43].

## Discussion

In this study, we focused on the availability of different sensory signals to the internal forward model that generates a prediction of a terminal movement outcome. So far, there is not much experimental research on the dependencies of the internal forward model on sensory signals from vision, proprioception, and audition or the efference copy as an efferent signal. In a recent study, we showed that action effect monitoring (i.e., observing the effect of the executed movement as it unfolds over time) was not an essential input for the prediction model (Joch et al., 2017). However, the absence of action effect monitoring led to a diminished amplitude of an ERP related to error prediction. In the present study, we further aimed to test the impact of visual and proprioceptive signals about the movement on prediction- and feedback-related ERPs. To do so, participants practiced a semi-virtual throwing task for four sessions (1600 trials total). The task in session 5 was to visually observe throwing movements (i.e., *visual* condition; no proprioceptive signals and no efference copy were available as inputs for the prediction model). In the sixth and last session, participants executed the motor task without any visual display of the effectors (i.e., condition *EffProp;* virtual lever and real arm of the participant were occluded). That way, the prediction model received no movement-related visual signals.

### Performance and Behavioral Results

**FIGURE 7:**
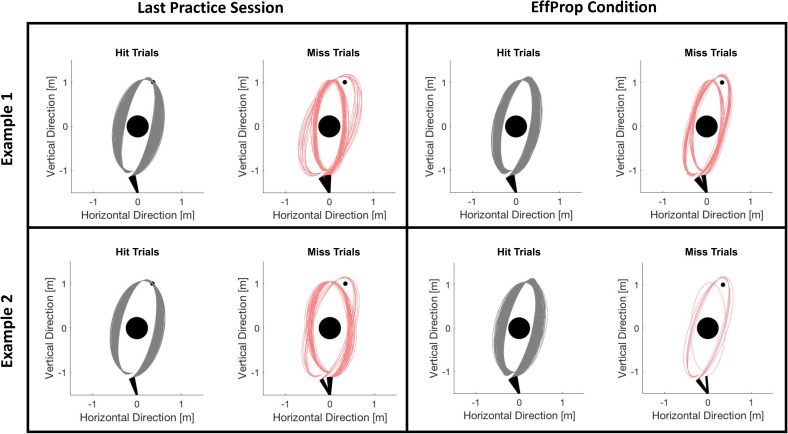
Comparison of ball flight trajectories of trials from session 4 (last practice session) and session 6 (*EffProp*) for two exemplary participants. The trials are separated in hit (gray) and error (red) trials. There was no change in strategies from session 4 to session 6.

Participants improved their task performance (quantified by the target hit rate in %) over the practice session from 65% to approximately 78%. Accounting for theoretical considerations by Jordan and Rumelhart (1992) and empirical findings from Maurer et al. (2015) and Joch et al. (2017), we assume that this performance increase reflects the development of an internal forward model during practice so that predicting the movement outcome was possible during the experimental conditions. Task performance dropped in session 5 (*Visual* condition) and decreased slightly further in session 6 (*EffProp* condition). However, hit rates during the experimental sessions were still similar to the hit rates described in previous studies using the Skittles task (Maurer et al., 2015; Joch et al., 2017). To ensure that the slight decreases did not arise from changes in throwing strategy (which could have an influence on the neural signals), we checked *post hoc* whether ball kinematics had changed from practice phase to the *EffProp* condition. For this, we plotted the trajectories of all trials executed in the last practice session and in the *EffProp* condition for each participant. We then manually checked for different throwing strategies. **Figure 7** exemplarily shows two participants. We could not find any strategy changes during *EffProp* in any of the participants.

### Electrophysiological Results – More Accurate Error Prediction With Visual Inputs

We quantified the accuracy of error prediction in two ways. First, by means of the Ne/ERN amplitude (i.e., high amplitude stands for high prediction accuracy and vice versa) and, second, using the complementary behavior between the Ne/ERN and FRN amplitudes (see section “Introduction”).

#### Ne/ERN Amplitude

We found a larger Ne/ERN mean amplitude (larger means more negative) in the *Visual* condition than in the *EffProp* condition. Hence, the reliability of the forward model predicting the throwing error seemed to be higher in the observation condition (neither efferent, nor proprioceptive input to the prediction model) compared to the execution condition without any visual input to the prediction model. The diminished effects of the Ne/ERN can be explained in terms of flexible strategies for sensory integration during motor planning (Sober and Sabes, 2005) suggesting a strong connection between the task’s target properties and the input requirements of the internal prediction model. Since the target in the Skittles task can be categorized as a visual target, it is possible that visual signals are essential during movement planning. Thus, restricting the heavily weighted visual signals in the *EffProp* condition might have led to high uncertainty of the outcome prediction and thus a miniscule amplitude of the Ne/ERN. On the other hand, in the *Visual* condition, where visual feedback was available but proprioceptive and efferent signals could not serve as inputs to the prediction model, the Ne/ERN indicated a more accurate error prediction. The smaller Ne/ERN in the *EffProp* condition could alternatively be explained by prediction error accounts (Holroyd and Coles, 2002; Alexander and Brown, 2011), which predict lower Ne/ERN amplitudes in conditions with more frequent errors and hence smaller prediction errors. With the present data we cannot finally rule out this possibility since the hit rate in the *EffProp* condition was in tendency (but not significantly) smaller than in the *Visual* condition. The reduced performance could either manifest in a lower error likelihood having an impact on error evaluation or in a poorer quality of the forward model corrupting error prediction. However, the performance difference of only 4% supports our belief that error likelihood should not noticeably deviate between conditions. Consequently, we assume that the prediction model in our goal-oriented throwing task relies more on visual signals.

Furthermore, the amplitude of the Ne/ERN in the *Visual* condition was smaller relative to conditions where visual and proprioceptive signals had been available (e.g., Joch et al., 2017). This is in line with studies investigating the so called “observation Ne/ERN” (e.g., Van Schie et al., 2004; Bates et al., 2005) that reported decreases in the Ne/ERN amplitude when participants observed the actions of another person.

#### FRN Amplitude and Ne/ERN – FRN Complementarity

Participants received a result-feedback in each trial of the practice and test sessions, informing them about target hit or miss 850 ms after ball release. In both, the *Visual* and *EffProp* conditions, we found a highly significant negative deflection within the effect window of 150–350 ms after the onset of outcome feedback. The amplitude of this FRN in the *EffProp* condition was, however, significantly more negative than in the *Visual* condition. Combining the observed neural responses related to error prediction (Ne/ERN) and outcome feedback (FRN), we find a complementary behavior of these two neural markers. In case of only limited sensory signals (efferent and proprioceptive signals or visual signals alone) being available to the system, the prediction model was not able to accurately predict the movement outcome and, thus, the corresponding neural correlate (Ne/ERN) was less pronounced (compared to the FRN) or absent. Subsequently, when the outcome feedback became available, presentation of an error gave rise to a strong FRN amplitude. This finding is in line with the observations of Weismüller and Bellebaum (2016) who suggest an association between the amplitude of the FRN and error awareness or error expectancy, respectively. Thus, since an error is less expected with a less accurate error prediction in the *EffProp* condition, the FRN is stronger after error occurrence. In the *Visual* condition, we observed the opposite behavior. The prediction model had access to visual signals during movement execution and was able to predict the movement outcome more accurately as in the *EffProp* condition, resulting in a stronger negative deflection of the Ne/ERN. Since the system was consequently aware of a possible upcoming error, the neural response to the result-feedback was less pronounced relative to the *EffProp* condition. Overall, the findings of Ne/ERN amplitude and FRN amplitude support the assumption that visual inputs are more important for error prediction in a goal-oriented throwing task.

### Limitations and Differences to Other Studies

The onset of the Ne/ERN potentials in our motor task is later than in studies using for example choice-reaction-time (CRT) tasks (e.g., Nieuwenhuis et al., 2004). This is not surprising since the throwing movements in the Skittles task are much more complex than button-presses in CRT tasks involving at least two joints (i.e., shoulder and elbow joint), whose sensory signals have to be evaluated and integrated together with visual signals to complement mere efferent information and yield a reliable estimation of the movement outcome. In our view, the longer onset latencies are a result of the longer processing time needed for the integration of these different information sources. Support for this interpretation comes from a study of Godefroid et al. (2016) who also found the Ne/ERN to be influenced by the number of sensory channels (Ne/ERN amplitude was modified by visual feedback in a Go/No-Go task, in addition to efferent information).

Furthermore, all of the observed mean amplitudes in EffW_ERN_ were smaller than the amplitudes within the same effect window and with same task as reported by Maurer et al. (2015) and Joch et al. (2017). However, these differences are in line with theoretical considerations as the quantity of input signals to the forward model was higher in the other two studies. In the study of Maurer and colleagues, the prediction model had access to a complete set of afferent and efferent input signals (efferent, visual, and proprioceptive signals of the movement as well as action effect monitoring). Joch and colleagues eliminated the action effect monitoring but kept all other afferent and efferent inputs of the movement. Thus, we assume that the mean amplitude of the Ne/ERN should attenuate with a decreasing prediction accuracy induced by restricted sensory input signals.

Another possible limitation of the present study arises from the fixed order of experimental sessions (session 5: *Vision*; session 6: *EffProp*). This procedure was chosen to provide a maximum of practice trials before executing the *EffProp* condition, which we expected to be the most difficult condition. Hence, task disengagement or fatigue could have led to poorer performance monitoring accompanied by a smaller Ne/ERN amplitude. However, the preserved and even larger amplitude of the FRN speak against this assumption.

## Conclusion

Altogether, the results of this study suggest that, at least in a goal-oriented throwing task like Skittles, visual signals about the movement are essential inputs to the internal prediction model. The prediction of an outcome error seems possible on the basis of visual signals alone. Conversely, restricting these signals might lead to a poorer prediction performance and less pronounced neural responses. However, the weighting of sensory signals could change, according to Sober and Sabes (2005), if a proprioceptive target is used in favor of proprioceptive signals, which could be an objective for a follow-up study. In situations where relevant inputs from sensory signals are not available for the model, prediction accuracy decreases and with it the occurrence (e.g., amplitude) of the Ne/ERN. Our findings are based on the effects of the two different experimental conditions (restricting visual signals vs. restricting efferent and proprioceptive signals) on the Ne/ERN and on the FRN. The two signals showed complementary behavior, which suggests that both brain potentials are related to the same motor error.

## Author Contributions

MJ and LKM conducted experiments, analyzed data, and drafted manuscript. MJ prepared figures. All authors interpreted results of experiments, edited, revised, and approved final version of manuscript and were involved in the conception and design of the study.

## Conflict of Interest Statement

The authors declare that the research was conducted in the absence of any commercial or financial relationships that could be construed as a potential conflict of interest.

## Supplementary Material

FIGURE S1 Grand Average EEG curves of all electrodes (black = hits, red = errors, blue = difference curve) -100 ms–400 ms around release for the *EffProp* condition. The green area marks the EffW_ERN_.

FIGURE S2 Grand Average EEG curves of all electrodes (black = hits, red = errors, blue = difference curve) -100 ms–400 ms around release for the *Visual* condition. The green area marks the EffW_ERN_.

FIGURE S3 Grand Average EEG curves of all electrodes (black = hits, red = errors, blue = difference curve) -100 ms–500 ms around feedback for the *EffProp* condition. The pink area marks the EffW_FRN_.

FIGURE S4 Grand Average EEG curves of all electrodes (black = hits, red = errors, blue = difference curve) -100 ms–500 ms around feedback for the *Visual* condition. The pink area marks the EffW_FRN_.
