# Supplementary figures and images for: Accuracy of Motor Error Predictions for Different Sensory Signals
Source: Front Psychol. 2018 Aug 7;9:1376. doi: 10.3389/fpsyg.2018.01376 (PMC6090479; doi:10.3389/fpsyg.2018.01376)

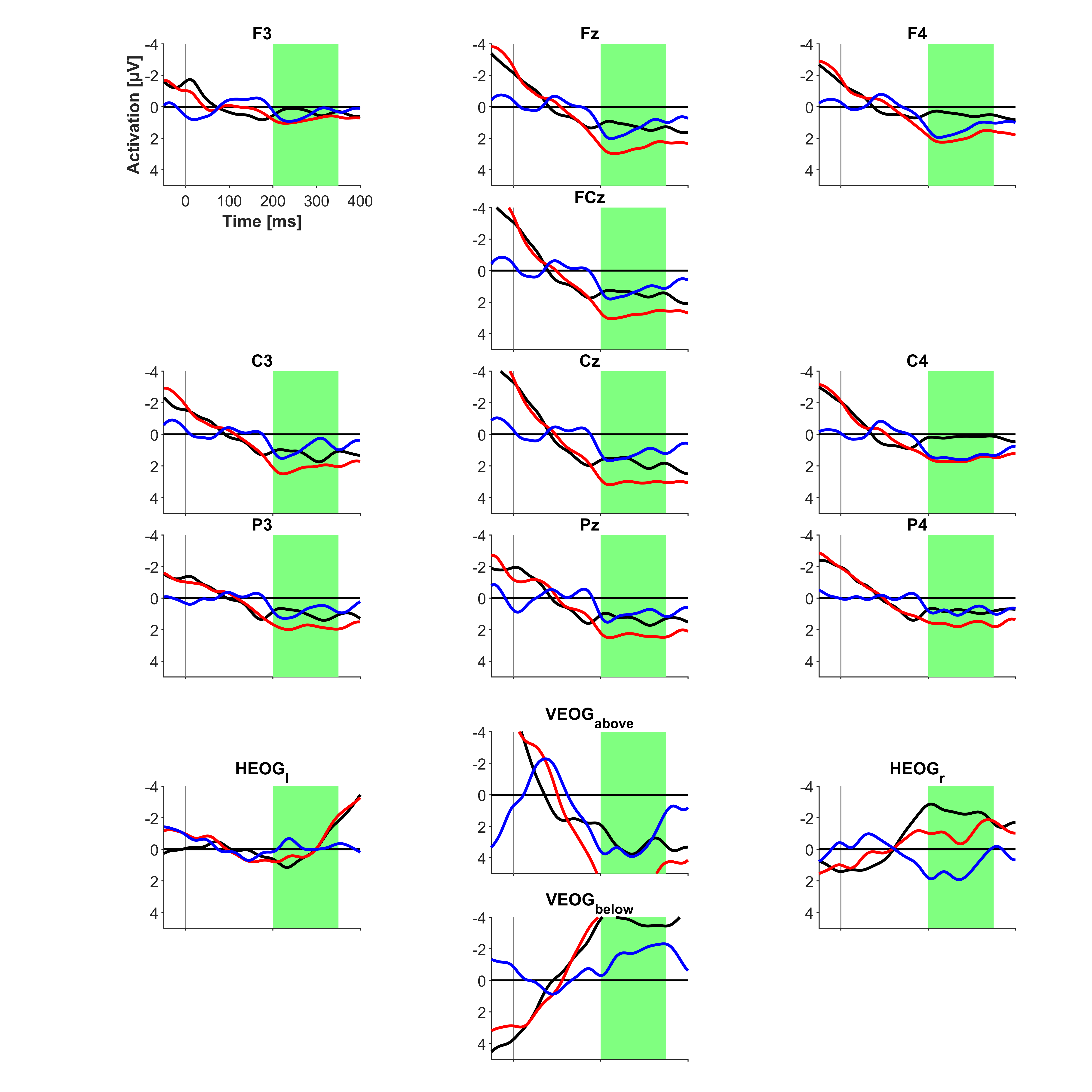

Supplement: FIGURE S1 — Grand Average EEG curves of all electrodes (black = hits, red = errors, blue = difference curve) -100 ms–400 ms around release for the EffProp condition. The green area marks the EffWERN. [file Image_1.TIF]

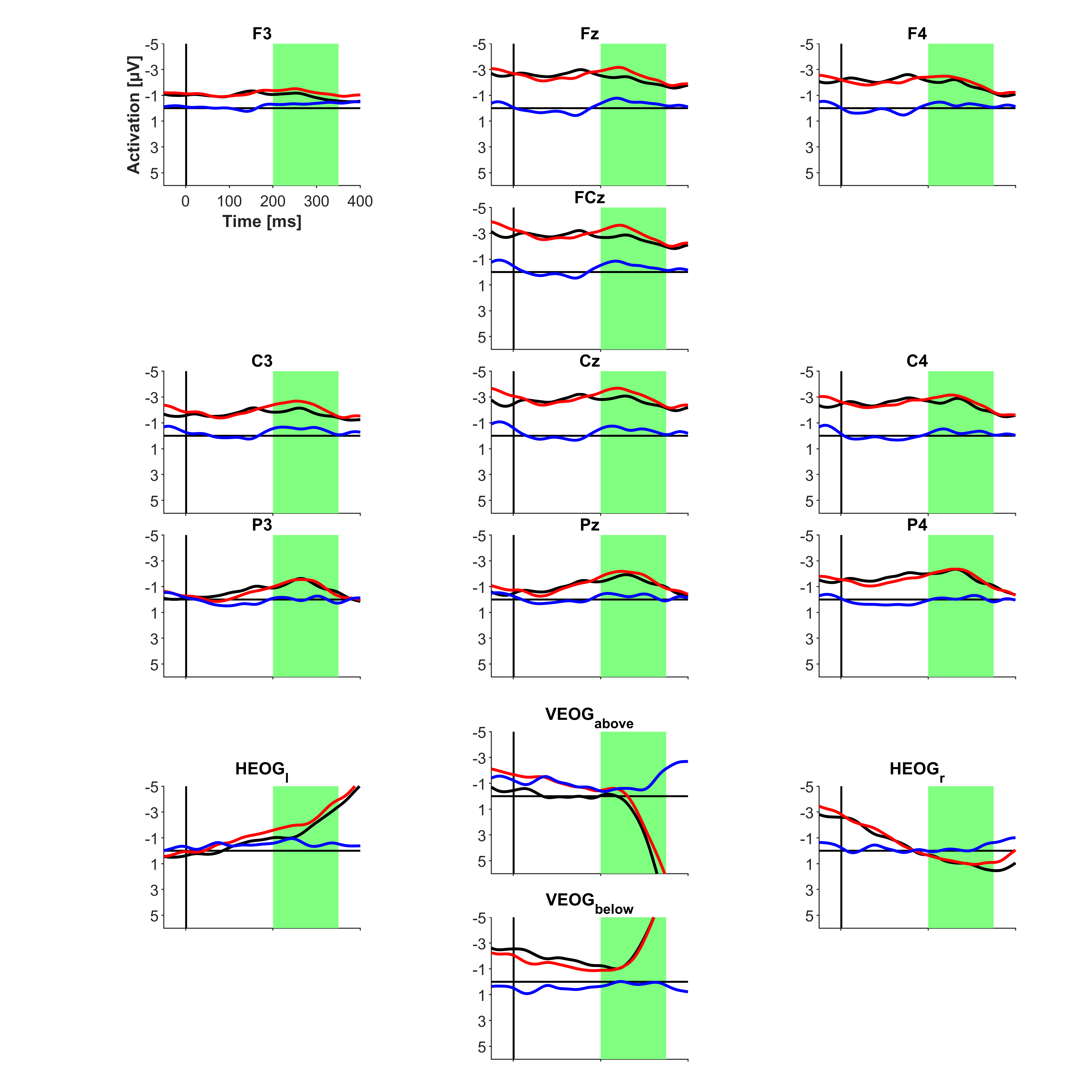

Supplement: FIGURE S2 — Grand Average EEG curves of all electrodes (black = hits, red = errors, blue = difference curve) -100 ms–400 ms around release for the Visual condition. The green area marks the EffWERN. [file Image_2.TIF]

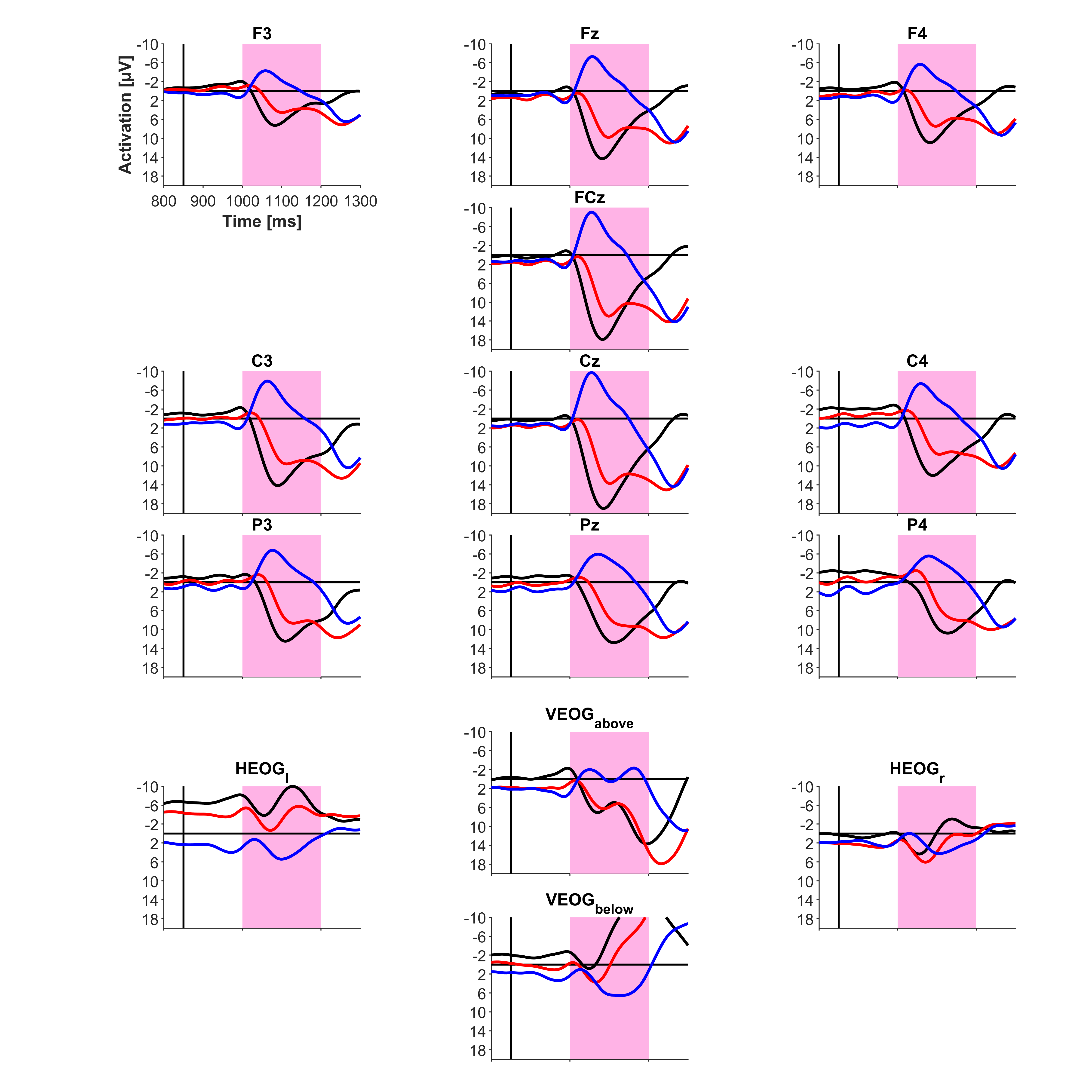

Supplement: FIGURE S3 — Grand Average EEG curves of all electrodes (black = hits, red = errors, blue = difference curve) -100 ms–500 ms around feedback for the EffProp condition. The pink area marks the EffWFRN. [file Image_3.TIF]

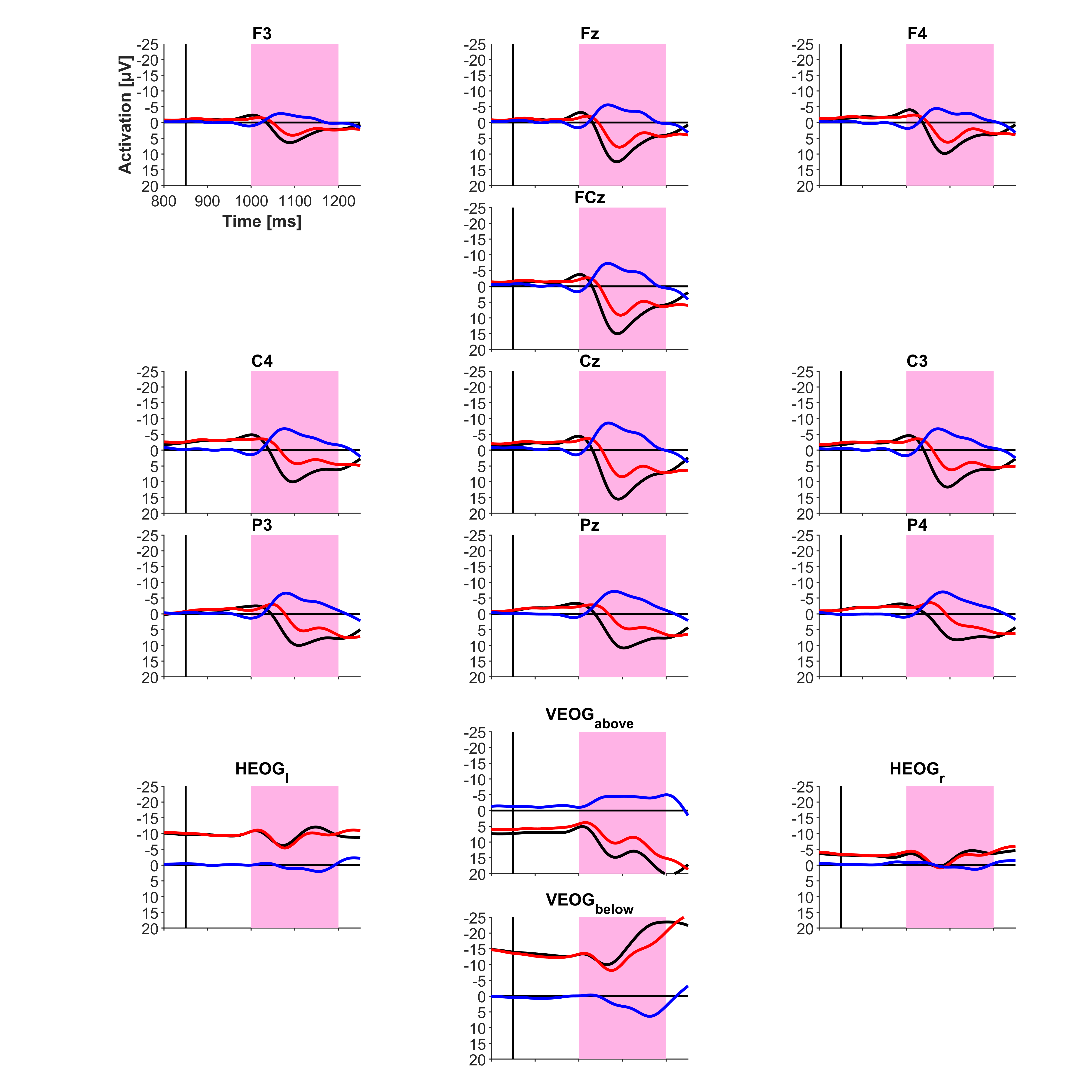

Supplement: FIGURE S4 — Grand Average EEG curves of all electrodes (black = hits, red = errors, blue = difference curve) -100 ms–500 ms around feedback for the Visual condition. The pink area marks the EffWFRN. [file Image_4.TIF]
